# Quality Improvement Leadership in Academic Children’s Hospitals

**DOI:** 10.1097/pq9.0000000000000034

**Published:** 2017-06-21

**Authors:** John A. Barnard, J. Terrance Davis

**Affiliations:** From the * Department of Pediatrics, The Ohio State University College of Medicine, Columbus, Ohio; † The Research Institute at Nationwide Children’s Hospital, Columbus, Ohio; ‡ Hospital Administration, Nationwide Children’s Hospital, Columbus, Ohio; § Department of Surgery, The Ohio State University College of Medicine, Columbus, Ohio; and ‖ Nationwide Children’s Hospital, Columbus, Ohio.

Children’s hospitals operate in a health care environment that is dramatically different from a decade ago. The public nature of the Institute of Medicine’s “Crossing the Quality Chasm”^1^ brought health care quality to hospital boards’ attention for the first time, and they mandated hospital leaders take prompt action to improve care quality and patient safety (PS). This focus on quality improvement (QI) and PS^2^ required hospital administrators, academic physicians, and nursing leadership to cooperate and collaborate to a much greater extent than they had traditionally. In addition, pressures on the business of health care required provider efficiency and productivity at levels not previously experienced in academic medicine. This, in turn, required administrators and physician leaders to work together in ways foreign to traditional academia. Evidence emerged suggesting that team science and team leadership was more effective than customary monolithic approaches to health care and academics.^3,4^ These external and internal forces have necessitated changes in emphasis and values that create challenges—and opportunities—for both academic and institutional leadership.

## CHALLENGES TO ACADEMIA

Academic leaders such as department chairs typically report to medical school deans. Together, they are responsible for faculty recruitment, training, research, and nurturing professional development to advance the science of medicine and provide high-quality education. The usual currency required for professional advancement in academia is publication of original peer-reviewed science and acquisition of grant funding. These are predominantly based on classical scientific method that differs in many ways from QI science.^2,5,6^ Additionally, the overall demand for QI and PS activity has created challenges for academic leaders.

The first challenge for academic leaders is the incorporation of QI and PS into the everyday fabric of the department’s activities. For example, residents and fellows, who deliver much of the care in the hospital setting, are crucial to any QI success,^7–9^ and department chairs must find ways to teach, evaluate, and reward QI activity in their trainees.^10^ Of course, this requires the requisite skill sets and bandwidth exist in the faculty to support that activity.

A related challenge is fully incorporating QI research and clinical activity into their department’s faculty culture. To do this, academic leaders must find ways to recognize QI activity in the algorithm for professional advancement up the promotion ladder.^11^ Currently, considerable variability exists in the criteria and processes for academic advancement related to QI activity. Consensus on appointment and promotion criteria and models, including publication requirements, would be useful. The potential for the development of a separate QI “track” is apparent^8,12^ and can even be incorporated into a medical schools promotion document.

A final challenge for academic leaders is to coordinate QI and PS activities among disparate divisions and departments. These activities are resource intense and must be considered in annual budgets and allocation of physician time away from more traditional revenue generating activities. Large departments, for example, pediatrics, may find QI and PS activities scalable, whereas smaller departments may be challenged by fewer resources and person power. In this case, the challenge is to find ways to provide the support necessary for smaller departments to generate meaningful and useful QI activity.

## CHALLENGES TO HOSPITAL ADMINISTRATION

Hospital administrators also experience challenges as they engage at the level of intensity needed for success in QI and PS.^13^ Senior hospital administrative leaders ultimately report to the Board of Directors. In the not-for-profit world, boards are comprised of talented, business-oriented, community leaders who may not have a medical background. Their business skills and community knowledge serve them well in focusing on high-level strategy, fiscal matters, and facilities. More recently, as boards have had to focus on QI and PS and the sometimes complex definitions and metrics related to quality and safety, their expertise has been challenged.

Other challenges to the relationship between hospital administrators and the board exist. For example, administrators must navigate how to present sometimes-negative information to the board in an understandable way, while assuring them that system issues are being properly addressed to prevent recurrence. Administrators must create an atmosphere in which transparency is the norm and failures and successes are discussed openly. It is widely understood that a transparent culture is necessary to make significant progress in QI and PS,^14,15^ but, depending on the nature of a hospital’s historical culture, this can be a multi-year journey.

Also, hospital administration must ensure sufficient infrastructure exists to support multiple simultaneous QI and PS projects.^16^ This includes not only a sufficient workforce in the QI Department, but sufficient information technology resources to support data demands. These are generally nonrevenue producing activities that compete with revenue producing staff positions and activities. One can make a good business case for quality work,^17^ but it may not be obvious at the outset and a natural tension between nonrevenue- and revenue-generating activities will always be a challenge for hospital leaders. In addition to providing resources, administration must provide the leadership to ensure that a common QI methodology is used and that centralized control and supervision of projects results in a coordinated and integrated system-wide QI process that is making measureable improvement.^18^

Finally, it falls to hospital administrators to provide basic training to all employees in high-reliability techniques. Improvement curricula designed to produce a significant cadre’ of individuals all trained in QI methodology have been used in institutions that have seriously taken on the challenges of QI and PS.^19,20^ In organizations where high reliability has become de rigueur, the benefits of the resultant efficiency and efficacy should be felt well beyond the domains of PS and QI.

## JOINT CHALLENGES

Given the significant challenges and the limited nature of available resources, systematic integration between academia and hospital administration is essential for optimal QI and PS outcomes.^21^ Clearly, multiple, independent, simultaneous QI projects in an academic children’s hospital system can be inefficient and nonproductive. A shared infrastructure utilized by all, with coordination and prioritization of projects in alignment with the hospital’s overall strategic plan is optimal. It is recognized that integration is a spectrum, and children’s hospitals are at various stages with respect to integration,^21^ but continual movement requires joint leadership from academics and administrators to be successful.

Another challenge is training of future leaders. Although many in leadership positions today got their training “on the job,” it is likely that formal training to learn appropriate skill sets will be necessary in the future. Hospital administrators will need to have training not only in business but also in QI and PS science. Conversely, physician leaders may need an Master of Business Administration (MBA) or Master of Healthcare Administration (MHA). Physicians working their way up the career ladder will need to be facile in both classic scientific method and QI science—being able to mentor and coach their faculty in both arenas. Recent requirements by Accreditation Council for Graduate Medical Education (ACGME) for trainees to have exposure to and participation in QI activities may set the stage for this. Fortunately, QI teaching is now finding a home in some medical school curricula.^22^

A final joint challenge is for academic leaders and hospital administration to find practical ways to create linkages and understand each other’s point of view. For example, inclusion of senior hospital administrators as “executive sponsors” in major QI Initiatives would be useful. Further, inclusion of academic leadership in discussions and decisions related to the periodic review of the hospital’s strategic planning process would further the necessary integration. Finally, make it a point to include authors from both groups in publications related to QI-related system changes.

## CONCLUSIONS

The academic and hospital administrative worlds are no longer separate entities. It is important for leaders in both areas to recognize that leadership in academic children’s hospitals is a team sport, and progress in QI and PS will happen most quickly and efficiently when both sides work together to accomplish mutually agreed upon goals.

## DISCLOSURE

The authors have no financial interest to declare in relation to the content of this article.
